# The Use of Digital Platforms for Adults’ and Adolescents’ Physical Activity During the COVID-19 Pandemic (Our Life at Home): Survey Study

**DOI:** 10.2196/23389

**Published:** 2021-02-01

**Authors:** Kate Parker, Riaz Uddin, Nicola D Ridgers, Helen Brown, Jenny Veitch, Jo Salmon, Anna Timperio, Shannon Sahlqvist, Samuel Cassar, Kim Toffoletti, Ralph Maddison, Lauren Arundell

**Affiliations:** 1 Institute for Physical Activity and Nutrition Deakin University Geelong Australia; 2 School of Humanities and Social Sciences Faculty of Arts and Education Deakin University Burwood Australia

**Keywords:** digital health, moderate- to vigorous-intensity physical activity, muscle-strengthening exercise, online platforms, COVID-19

## Abstract

**Background:**

Government responses to managing the COVID-19 pandemic may have impacted the way individuals were able to engage in physical activity. Digital platforms are a promising way to support physical activity levels and may have provided an alternative for people to maintain their activity while at home.

**Objective:**

This study aimed to examine associations between the use of digital platforms and adherence to the physical activity guidelines among Australian adults and adolescents during the COVID-19 stay-at-home restrictions in April and May 2020.

**Methods:**

A national online survey was distributed in May 2020. Participants included 1188 adults (mean age 37.4 years, SD 15.1; 980/1188, 82.5% female) and 963 adolescents (mean age 16.2 years, SD 1.2; 685/963, 71.1% female). Participants reported demographic characteristics, use of digital platforms for physical activity over the previous month, and adherence to moderate- to vigorous-intensity physical activity (MVPA) and muscle-strengthening exercise (MSE) guidelines. Multilevel logistic regression models examined differences in guideline adherence between those who used digital platforms (ie, users) to support their physical activity compared to those who did not (ie, nonusers).

**Results:**

Digital platforms include streaming services for exercise (eg, YouTube, Instagram, and Facebook); subscriber fitness programs, via an app or online (eg, Centr and MyFitnessPal); facilitated online live or recorded classes, via platforms such as Zoom (eg, dance, sport training, and fitness class); sport- or activity-specific apps designed by sporting organizations for participants to keep up their skills (eg, TeamBuildr); active electronic games (eg, Xbox Kinect); and/or online or digital training or racing platforms (eg, Zwift, FullGaz, and Rouvy). Overall, 39.5% (469/1188) of adults and 26.5% (255/963) of adolescents reported using digital platforms for physical activity. Among adults, MVPA (odds ratio [OR] 2.0, 95% CI 1.5-2.7), MSE (OR 3.3, 95% CI 2.5-4.5), and combined (OR 2.7, 95% CI 2.0-3.8) guideline adherence were higher among digital platform users relative to nonusers. Adolescents’ MVPA (OR 2.4, 95% CI 1.3-4.3), MSE (OR 3.1, 95% CI 2.1-4.4), and combined (OR 4.3, 95% CI 2.1-9.0) guideline adherence were also higher among users of digital platforms relative to nonusers.

**Conclusions:**

Digital platform users were more likely than nonusers to meet MVPA and MSE guidelines during the COVID-19 stay-at-home restrictions in April and May 2020. Digital platforms may play a critical role in helping to support physical activity engagement when access to facilities or opportunities for physical activity outside the home are restricted.

## Introduction

Physical activity plays an important role in the prevention and treatment of noncommunicable diseases, which account for 70% of deaths worldwide [1]. A recent study of 168 countries estimated that 3.9 million (15%) premature deaths could be averted annually if more people engaged in recommended levels of physical activity [2]. The World Health Organization recommends that, each week, adults should accumulate at least 150 minutes of moderate-intensity physical activity, 75 minutes of vigorous-intensity physical activity, or an equivalent combination of moderate- to vigorous-intensity physical activity (MVPA); in addition, youth aged 5 to 17 years should accumulate at least 60 minutes of MVPA daily [3]. Adults are also advised to perform muscle-strengthening exercises (MSEs) at least twice per week, and adolescents are advised to perform them at least three times per week [3]. The guidelines are consistent with those in Australia [4,5]. Globally, 19% of adolescents [6] and 73% of adults achieve the MVPA guidelines according to the most recent estimates [7]. National survey data from 2017-2018 show that fewer Australians engaged in the recommended physical activity levels than the global average, with 55% of adults (18-64 years) and 10% of adolescents (15-17 years) achieving the recommended MVPA [8], and 1 in 6 adults (15%) and adolescents (16%) adhering to the MSE guidelines [8]. These prevalence data have been observed in the general population under free-living conditions. However, conditions have changed considerably as a result of government responses to COVID-19, which led to unprecedented and widespread social distancing measures to control its spread [9].

In Australia, for example, the federal government announced strict *stay-at-home* orders in late March 2020. Although the length of these restrictions varied by state and territory, opportunities to perform some physical activities outside the home, such as at gyms and sport clubs, were impacted nationwide [9,10]. Google Trends data showed that online queries of how to perform physical activity and exercise peaked in Australia during the first 2 weeks that the stay-at-home restrictions were imposed [11]. While this shows that people were investigating ways to keep active during this period, it does not provide information on the sort of support or the types of programs people may have used to be active during this time. Over the same period, there was an exponential increase in the use of the internet and associated digital platforms, such as websites and smartphone apps, as they became essential for education, work, and social interactions [12]. Digital platforms have previously shown promise for increasing physical activity among individuals of all ages in intervention studies [13-15]. However, use among Australians prior to the pandemic was low. Surveys of Australian adults (≥15 years of age) in 2018 showed that just 18.7% of adults used apps for tracking activity or training, and engagement with websites or online tools (7.1%) and online videos for sport (2.5%) was low [16].

Understanding the types of digital platforms used during the unprecedented pandemic situation could provide insight into their potential role for supporting individuals in meeting MVPA, MSE, and combined physical activity recommendations when individuals are unable to access traditional physical activity settings and facilities. This study aimed to explore the use of digital platforms for physical activity in Australia during April and May 2020 and to examine associations between the use of digital platforms and adherence to physical activity guidelines among adults and adolescents.

## Methods

### Overview

Data were drawn from the baseline sample of the Our Life at Home study (OL@H), collected May 4-31, 2020. OL@H is a longitudinal study designed to investigate the impact of the Australian Governments’ response to managing the COVID-19 pandemic on movement behaviors and on the health and well-being of Australians aged 13 to 75 years over a 2-year period. OL@H received ethical approval from the Deakin University Human Ethics Advisory Group-Health (HEAG-H 59_2020).

### Context

**Figure 1:**
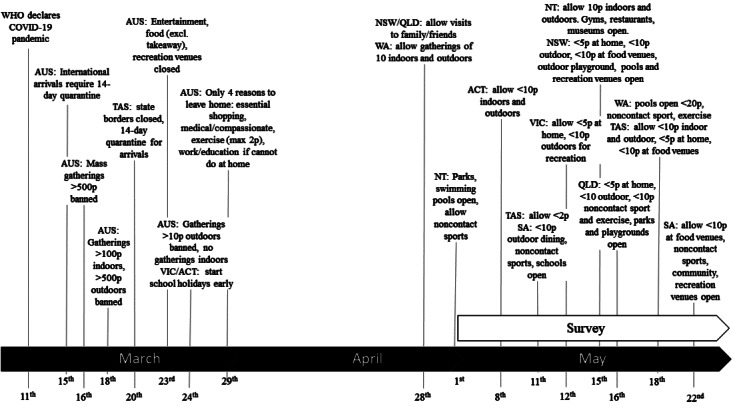
Australian federal- and state-imposed restrictions to stop the spread of COVID-19. ACT: Australian Capital Territory; AUS: Australia; NSW: New South Wales; NT: Northern Territory; p: people; QLD: Queensland; SA: South Australia; TAS: Tasmania; VIC: Victoria; WA: Western Australia; WHO: World Health Organization.

From March 29, 2020, strict stay-at-home orders were imposed by the Australian Government. As a result, all organized and social sports were suspended, and gyms and recreation facilities temporarily closed. People were allowed to leave their home to exercise as long as they maintained a 1.5-meter distance from those not living in their household. Each state and territory had the power to decide on and enforce their own restrictions, with several states easing restrictions on organized sport and recreation facilities in early May due to no or low recorded cases of COVID-19 (see Figure 1).

### Sample

Individuals living in Australia were recruited via social media advertising (eg, Facebook and Instagram), researcher and stakeholder organization networks, and snowballing techniques, wherein participants were asked to share the study information with others they knew (eg, word of mouth). Of 14,764 individuals who clicked on the link to the survey, 6474 provided informed consent (43.8% response rate), of which 4079 (63.0%) were adolescents (13-17 years) and 2395 (37.0%) were adults (18-75 years).

### Measures

#### Demographic Characteristics

Participants reported their age (years), sex (male, female, other, or prefer not to say), usual daily responsibilities (paid employment status, home duties or carer responsibilities, and student status; yes or no), number of people living in the household, whether the English language was spoken at home (yes or no), and state or territory of residence.

#### Adherence to the Physical Activity Guidelines

Adherence to physical activity guidelines was assessed using valid and reliable survey items [17-19]. Participants were asked to indicate how many days per week (none, 1 day/week, 2 days/week, 3 days/week, 4 days/week, 5 days/week, 6 days/week, or 7 days/week) they performed MVPA during a usual week over the past month (ie, April or May 2020) and in February 2020 (ie, pre-COVID-19 restrictions) for 30 minutes (adults aged ≥18 years) or 60 minutes (adolescents aged 13-17 years). Adult responses were dichotomized at 5 or more days per week (ie, ≥150 minutes/week), and adolescent responses were dichotomized at 7 days per week (ie, ≥60 minutes/day). Participants were also asked whether they performed (yes or no) MSE during a usual week (at home over the past month [April or May] or at home or at a gym during February); those who said *yes* were then asked to report the number of times per week (ie, frequency) that they performed MSE during a usual week. This was used to determine adherence to the MSE guidelines (adolescents: ≥3 times/week; adults: ≥2 times/week). Adherence to the combined guidelines was also calculated for the past month (April or May) and February.

#### Digital Platforms for Physical Activity

Participants were asked to respond to the question “Are you currently doing any form of sports or physical activities using online or digital platforms to assist or guide your activity?” (yes or no). A comprehensive list of digital platforms was identified in collaboration with key stakeholders. Participants who responded *yes* were asked to report the frequency (number of times per week) and duration (total minutes per week) spent using each of six types of online or digital platforms: streaming services for exercise (eg, YouTube, Instagram, and Facebook); subscriber fitness programs, via an app or online (eg, Centr and MyFitnessPal); facilitated online live or recorded classes, via platforms such as Zoom (eg, dance, sport training, and fitness class); sport- or activity-specific apps designed by sporting organizations for participants to keep up their skills (eg, TeamBuildr); active electronic games (eg, Xbox Kinect); and/or online or digital training or racing platforms (eg, Zwift, FullGaz, and Rouvy).

### Statistical Analysis

Demographic characteristics, physical activity, and digital platform use were presented descriptively. Demographic differences between those who used digital platforms (ie, users) compared to those who did not (ie, nonusers) were calculated using chi-square tests and *t* tests. Unadjusted logistic regression models were used to identify associations between sample characteristics (ie, age, sex, English-speaking household, number of people in household, employment status, home duties or carer responsibilities, and student status) and MVPA, MSE, and combined physical activity guideline adherence. Adjusted multilevel logistic regression models were then run to examine associations between the use of digital platforms and adherence with MVPA, MSE, and both MVPA and MSE guidelines. All multilevel models accounted for clustering by state or territory of residence and were adjusted for sample characteristics found to be significant in univariate models (see Multimedia Appendix 1) and guideline adherence during February 2020. Analyses were stratified by age group (ie, adults and adolescents). All analyses were performed using Stata v16 (StataCorp LLC).

## Results

Table 1 presents the sample characteristics for adult and adolescent participants. In total, 1188 adults and 963 adolescents provided complete physical activity guideline adherence and digital platform data and were included in analyses. Among adults, the mean age was 37.4 (SD 15.1) years, the majority were female, and two-thirds had a tertiary degree. In the past month, 33.0% (392/1188), 37.3% (443/1188), and 17.7% (210/1188) of adults met the MVPA, MSE, and both guidelines, respectively, and 39.5% (469/1188) used online or digital platforms to assist or guide their physical activity. Among those who used digital platforms for physical activity, the median frequency was 4 (IQR 2-6) times per week and the median duration was 105 (IQR 60-180) minutes per week. Among adolescents, the mean age was 16.2 (SD 1.2) years, and more than two-thirds were female. In the past month, 7.2% (69/963), 28.1% (271/963), and 3.6% (35/963) of adolescents met the MVPA, MSE, and both guidelines, respectively, and 26.5% (255/963) used online or digital platforms to guide or assist their physical activity. Among those who used digital platforms for physical activity, the median frequency was 4 (IQR 3-7) times per week and the median duration was 120 (IQR 60-260) minutes per week.

**Table 1:** Sample characteristics.

Characteristic		Adults (n=1188)	Adolescents (n=963)
Age in years, mean (SD)		37.4 (15.1)	16.2 (1.2)
Sex (female), n (%)		980 (82.5)	685 (71.1)
Employment status (working), n (%)		625 (52.6)	248 (25.8)
Home duties or carer responsibilities (yes), n (%)		180 (15.2)	127 (13.2)
Student status (yes), n (%)		258 (21.7)	622 (64.6)
Number of people in household, mean (SD)		3.2 (1.4)	4.3 (1.3)
English-speaking household (yes), n (%)		1155 (97.2)	931 (96.7)
**State or territory of residence, n (%)**			
	Australian Capital Territory	47 (4.0)	23 (2.4)
	New South Wales	217 (18.3)	234 (24.3)
	Northern Territory	9 (0.8)	4 (0.4)
	Queensland	124 (10.4)	197 (20.5)
	South Australia	79 (6.6)	71 (7.4)
	Tasmania	50 (4.2)	47 (4.9)
	Victoria	590 (49.7)	305 (31.7)
	Western Australia	72 (6.1)	82 (8.5)

Table 2 presents the demographic characteristics of digital platform users and nonusers. Among adults, a higher percentage of users were female and working in paid employment, and a higher percentage of nonusers had home duties or carer responsibilities. Among adolescents, a higher percentage of users were female.

**Table 2:** Demographic characteristics of users and nonusers of digital platforms for physical activity.

Characteristic	Adults (n=1188)				Adolescents (n=963)		
	Users	Nonusers	*P* value	Users		Nonusers	*P* value
Number of participants out of total, n (%)	469 (39.5)	719 (60.5)	N/A^a^	255 (26.5)		708 (73.5)	N/A
Age in years, mean (SD)	36.3 (13.2)	38.0 (16.2)	.06	16.2 (1.3)		16.3 (1.2)	.22
Sex (female), n (%)	428 (91.3)	552 (76.8)	<.001	213 (83.5)		472 (66.7)	<.001
Employment status (working), n (%)	281 (59.9)	344 (47.8)	<.001	63 (24.7)		185 (26.1)	.66
Home duties or carer responsibilities (yes), n (%)	56 (11.9)	124 (17.3)	.01	40 (15.7)		87 (12.3)	.17
Student status (yes), n (%)	89 (19.0)	169 (23.5)	.06	166 (65.1)		456 (64.4)	.84
English-speaking household (yes), n (%)	453 (96.6)	702 (97.6)	.28	245 (96.1)		686 (96.9)	.53
Number of people in household, mean (SD)	3.1 (1.3)	3.2 (1.5)	.56	4.4 (1.3)		4.3 (1.3)	.13

^a^N/A: not applicable; *P* values were not calculated for this item.

**Figure 2:**
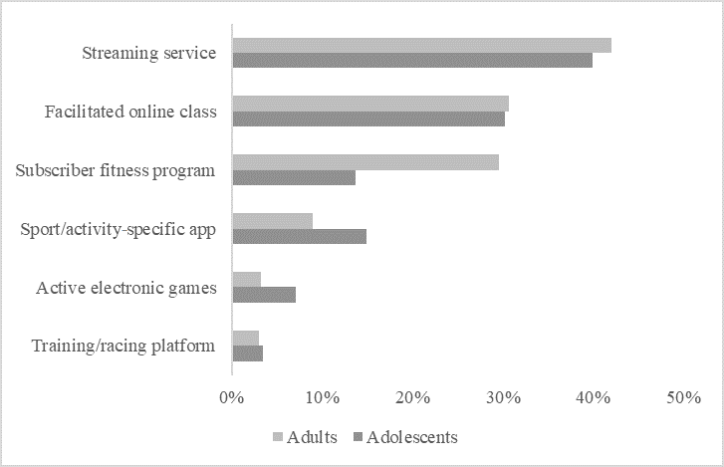
Use of each type of digital platform for physical activity among users.

Among those who had used digital platforms to guide or assist their physical activity, the most common were streaming services (adults: 197/469, 42.0%; adolescents: 102/255, 40.0%), facilitated online classes (adults: 144/469, 30.7%; adolescents: 77/255, 30.2%), and subscriber fitness programs (adults: 139/469, 29.6%; adolescents: 35/255, 13.7%) (see Figure 2). The types of digital platforms used were generally similar for adults and adolescents; however, proportionally fewer adolescents used subscriber fitness programs.

Table 3 presents the unadjusted and adjusted odds ratios for physical activity guideline adherence, accounting for state or territory of residence and adjusting for significant confounders (see Table S1 in Multimedia Appendix 1) and guideline adherence during February. In the adjusted models, adults who used digital platforms had 2 times the odds of meeting MVPA guidelines, over 3 times the odds of meeting MSE guidelines, and almost 3 times the odds of meeting the combined guidelines compared to nonusers. Adolescents who used digital platforms had more than 2 times the odds of meeting MVPA guidelines, over 3 times the odds of meeting MSE guidelines, and more than 4 times the odds of meeting the combined guidelines compared to nonusers.

**Table 3:** Adjusted odds ratios (ORs) for the associations of physical activity guideline adherence and use of digital platforms.

Model	Digital platform use for adults, OR (95% CI)^a^			Digital platform use for adolescents, OR (95% CI)^a^		
	MVPA^b^	MSE^c^	Combined	MVPA	MSE	Combined
Unadjusted model (reference: nonusers)	1.97 (1.54-2.52)	3.79 (2.96-4.87)	3.22 (2.36-4.39)	2.43 (1.47-3.99)	3.09 (2.27-4.20)	5.05 (2.50-10.18)
Adjusted model (reference: nonusers)	1.99 (1.49-2.66)^d^	3.34 (2.49- 4.47)^e^	2.73 (1.95-3.81)^f^	2.40 (1.32-4.35)^g^	3.07 (2.12-4.44)^h^	4.32 (2.09-8.95)^i^

^a^All accounted for clustering by state or territory and were adjusted for February guideline adherence.

^b^MVPA: moderate- to vigorous-intensity physical activity.

^c^MSE: muscle-strengthening exercise.

^d^Additionally adjusted for age, number of people living in household, home duties or carer responsibilities, and student status.

^e^Additionally adjusted for employment and home duties or carer responsibilities.

^f^Additionally adjusted for home duties or carer responsibilities.

^g^Additionally adjusted for student status.

^h^Additionally adjusted for sex and home duties or carer responsibilities.

^i^Additionally adjusted for home duties or carer responsibilities.

## Discussion

### Principal Findings

Findings from this study showed that digital platforms may play a critical role in supporting physical activity engagement during times when people have limited access to traditional settings or opportunities for physical activity outside the home. Individuals who used a digital platform were more likely to report achieving recommended levels of MVPA and MSE during April and May when strict stay-at-home orders were imposed for most of the nation.

Data sourced from the Global Digital Overview in January 2020 highlighted the ubiquitous use of digital platforms in Australia; 88% of the adult population had access to the internet, 71% used social media (eg, Facebook and Instagram), and 26% of internet users reported using health and fitness apps [20]. While there are no directly comparable data, data from our study suggest that a higher proportion of Australians used digital platforms for physical activity during April and May 2020 compared to 2018 [16], with a higher rate of use observed among adults compared with adolescents. In addition to the established use of mobile fitness programs [21], our findings suggest that people also used digital platforms for facilitated online live or recorded activity classes and for streaming services.

More females than males used digital platforms to guide or assist their physical activity, which is similar to previous research [22,23]. This may reflect the ability of digital platforms to support information sharing, self-monitoring, and internal accountability, which are often considered important for increasing physical activity motivation among females [24,25]. It may be that females participate more in instructor-led activities (eg, yoga, Pilates, and dance) [26], whereas males tend to engage in more organized sport and weight training [8], both of which are less adaptable to online delivery via digital platforms. Safety concerns when exercising alone outdoors or after dark and fear of judgement are known barriers to physical activity uptake by women [27,28]; this may have also informed women’s decisions to use digital platforms to undertake physical activity in the home. Alternatives such as digital platforms may play an important role in ensuring that women achieve sufficient physical activity levels. Digital platforms can be used at any time of the day and offer convenience of use in the home, which may explain the larger proportion of adults working in paid employment using digital platforms for physical activity compared to those not working.

In this sample, 33% of adults reported meeting MVPA guidelines during the April and May stay-at-home period, which is considerably lower than the Australian average of 55% of 18- to 64-year-olds in 2017-2018 [8]. It should be noted that the MVPA measure in this study required adults to engage in 30 minutes per day of MVPA on at least 5 days per week, whereas the Australian Bureau of Statistics physical activity measure was based on a minimum total of 150 minutes of moderate-intensity physical activity, 75 minutes of vigorous-intensity physical activity, or an equivalent combination per week [8]. Among adolescents, just 7% met guidelines for MVPA, which was slightly lower than the Australian average of 10% of 15- to 17-year-olds [8]. These differences may have been due to the reduced ability to access traditional settings for physical activity, such as schools [29], work [30], and fitness and recreation facilities [31], or to participate in sport or active travel [32]. In contrast, the proportion of adults and adolescents in this sample who met the MSE guidelines during the April and May stay-at-home period was considerably higher than the Australian average [8]. MSE includes bodyweight activities that may not require specialized equipment or facilities; can be performed in a confined space, such as at home; and may have been promoted via digital platforms (eg, livestreams on YouTube) during the stay-at-home restriction phase.

Our results showed support for the use of digital platforms to engage in physical activity and MSE when access to traditional settings and facilities for physical activity was restricted. Adults and adolescents who used digital platforms to guide or assist physical activity were more likely to meet the MVPA, MSE, and combined physical activity guidelines compared to those who did not use digital platforms. This is consistent with evidence from Germany [33] and the United States [22] that showed that adults who used physical activity and health apps engaged in more physical activity compared to those who did not. This study builds on the evidence that digital technologies can promote and support physical activity among adults and adolescents [13,34-39]. Streaming services were the most frequently used digital platform to guide or assist physical activity, so future studies should further explore how they can best support physical activity engagement for all demographic groups and how they can be used as a physical activity promotion tool. Streaming services are mostly free to use and, thus, present an opportunity for relevant government or nongovernment organizations to make use of this platform for education, instruction, and promotion of physical activity to the general public.

Overall, the findings highlight a willingness to engage with technology for MVPA and MSE when access to traditional settings or opportunities for physical activity outside the home are limited, particularly among females and working adults. Further research is needed to explore what motivated or discouraged people from using digital platforms during this period of reduced options for activity outside the home environment. Future work could also explore whether the use of digital platforms for physical activity replaced usual physical activity behaviors from before the restrictions or complemented other physical activities (eg, attending a fitness class in person) and how digital platforms can best support continued engagement in physical activity once restrictions are reduced.

### Limitations

The large sample size and measures of MVPA, MSE, and combined guideline adherence are strengths of this study. However, the majority of participants were female and English speaking; in addition, 50% of adults and 32% of adolescents were from one state—Victoria—and, thus, are not representative of the wider Australian population. As the survey was completed online, English-language proficiency was required, which may have also reduced the generalizability of the findings to culturally and linguistically diverse populations. The measure used to capture MVPA guideline adherence in this study was valid and reliable [17-19]; however, it may not accurately capture individuals who engaged in shorter durations of vigorous-intensity physical activity yet still met the guidelines (eg, 30 minutes/day and 3 days/week). Participants were asked to report on MSE specifically in the home during April and May and at home or a gym in February; as such, this may not have captured all MSE performed (eg, in other locations). In addition, this study relied on self-report of physical activity and, as such, potential for recall bias must be acknowledged.

### Conclusions

In this study, fewer than half of the adults and one-third of adolescents reported using digital platforms to assist or guide their physical activity during the COVID-19 stay-at-home period in April and May 2020. Both adults and adolescents who used digital platforms for physical activity were more likely to meet the MVPA, MSE, and combined physical activity guidelines compared to those who did not use digital platforms. This suggests that digital platforms can play a critical role in supporting physical activity engagement. There is a need for future research to understand sustained use, gender preferences, and motivations for the use of digital platforms to guide or assist physical activity, in particular via streaming services, given their popularity during COVID-19.

## Appendix

Multimedia Appendix 1 Unadjusted odds ratios (95% CI) for the associations of sample characteristics and physical activity guideline adherence in April and May 2020.

## Abbreviations

MSE: muscle-strengthening exercise
MVPA: moderate- to vigorous-intensity physical activity
OL@H: Our Life at Home study
