# Supplementary material for: The Use of Digital Platforms for Adults’ and Adolescents’ Physical Activity During the COVID-19 Pandemic (Our Life at Home): Survey Study
Source: J Med Internet Res. 2021 Feb 1;23(2):e23389. doi: 10.2196/23389 (PMC7857525; doi:10.2196/23389)
Supplement: Multimedia Appendix 1 [file jmir_v23i2e23389_app1.docx]

Table S1. Unadjusted odds ratios (95% CI) for the associations of sample characteristics and physical activity guideline adherence in April/May 2020

|  | Adults | | | Adolescents | | |
| --- | --- | --- | --- | --- | --- | --- |
|  | MVPA | MSE | Combined | MVPA | MSE | Combined |
| Age | 1.03 (1.02-1.03) | 1.00 (0.99-1.00) | 1.01 (0.99-1.02) | 0.84 (0.70-1.02) | 1.02 (0.91-1.15) | 1.13 (0.84-1.50) |
| Sex (ref: male) | 0.78 (0.58-1.03) | 1.11 (0.84-1.47) | 0.83 (0.59-1.18) | 1.14 (0.74-1.76) | 0.74 (0.57-0.97) | 1.61 (0.93-2.78) |
| English speaking household (ref: no) | 2.26 (0.92-5.52) | 0.88 (0.43-1.81) | 1.57 (0.55-4.53) | 2.37 (0.32-17.66) | 0.92 (0.41-2.05) | ^a^ |
| Number of people in household | 0.91 (0.84-0.99) | 1.02 (0.94-1.11) | 0.94 (0.85-1.05) | 1.08 (0.90-1.31) | 1.04 (0.93-1.16) | 1.14 (0.88-1.47) |
| Employment status (ref: not working) | 1.11 (0.87-1.42) | 1.30 (1.02-1.65) | 1.01 (0.75-1.36) | 0.79 (0.44-1.42) | 0.83 (0.60-1.15) | 1.16 (0.55-2.45) |
| Home duties/carer responsibilities (ref: no) | 0.56 (0.38-0.81) | 0.63 (0.44-0.88) | 0.44 (0.26-0.74) | 1.58 (0.84-2.98) | 1.66 (1.12-2.47) | 2.019(0.88-4.54) |
| Student status (ref: not studying) | 0.52 (0.38-0.72) | 0.95 (0.72-1.27) | 0.70 (0.48-1.04) | 0.61 (0.37-0.99) | 0.95 (0.71-1.28) | 0.82 (0.41-1.63) |

^a^All participants meeting both guidelines were from English speaking households, therefore the model could not be run.

MVPA: moderate-to-vigorous intensity physical activity; MSE: muscle strengthening exercise
